# Task demands, tDCS intensity, and the COMT val^158^met polymorphism impact tDCS-linked working memory training gains

**DOI:** 10.1038/s41598-017-14030-7

**Published:** 2017-10-18

**Authors:** Jaclyn A. Stephens, Kevin T. Jones, Marian E. Berryhill

**Affiliations:** 1 University of Nevada, Department of Psychology, Program in Cognitive and Brain Sciences, Reno, Nevada USA; 2 Kennedy Krieger Institute, Department of Physical Medicine and Rehabilitation Baltimore, Maryland, USA; 3 Johns Hopkins School of Medicine, Department of Physical Medicine and Rehabilitation, Baltimore, Maryland, USA; 4 Colorado State University, Department of Occupational Therapy, Fort Collins, Colorado, USA; 5 Colorado State University, Department of Psychology, Fort Collins, Colorado, USA

## Abstract

Working memory (WM) training paired with transcranial direct current stimulation (tDCS) can improve executive function in older adults. The unclear mechanism of tDCS likely depends on tDCS intensity, and task relevant genetic factors (e.g., for WM: COMT val^158^met, DAT, BDNF val^66^met). Higher tDCS intensity does not always lead to greater cognitive gains, and genetic polymorphisms may modulate tDCS-linked WM improvements. To evaluate these factors, 137 healthy older adults provided DNA samples and received Visual and Spatial WM training paired with tDCS (sham, 1, 1.5, 2 mA). After one session of tDCS, significant group differences in WM performance were predicted by COMT val^158^met status. One month after training, there was a significant interaction of tDCS intensity, COMT genotype, and WM task. Specifically, val/val homozygotes benefited most from 1.5 mA tDCS on Visual WM and from 1 mA tDCS on Spatial WM. For met/met homozygotes, 2 mA resulted in significantly poorer performance compared to 1.5 mA on Spatial WM. While this pattern was observed with relatively small sample sizes, these data indicate that variations in COMT val^158^met may predict the nature of WM improvement after initial and longitudinal tDCS. This contributes to our understanding of the underlying mechanism by which tDCS affects behaviour.

## Introduction

Working memory (WM) is essential for most cognitive tasks, yet is capacity limited, declines with age, and resists improvement. The importance of WM has prompted an industry devoted to enhancing WM, despite mixed evidence of efficacy^1,2^. One promising approach involves pairing WM training with non-invasive brain stimulation, including transcranial direct current stimulation (tDCS)^3^. Several longitudinal WM training + tDCS studies consistently show improvement in trained tasks^4–6^ and some even report transfer to untrained WM tasks^7–12^. Unfortunately, there is no comprehensive understanding of the mechanism underlying tDCS effects on behaviour. This gap in knowledge limits the translational potential of tDCS. What is known is that tDCS alters synaptic plasticity across many neurotransmitters and neuromodulators (reviewed in:^13–16^). At a network level, tDCS strengthens functional connectivity^6,17–30^ and enhances neural synchrony^31^. Importantly, factors such as educational attainment^32^, WM capacity^33^, and motivation^34^ also significantly influence responses to the same WM-tDCS protocol. Because such factors predict different responses to a single tDCS protocol, averaging across heterogeneous participants obscures tDCS effects. This likely contributes to inconsistent findings and may explain recent meta-analyses challenging the usefulness of tDCS^35–39^.

Additionally, the mechanism of tDCS likely differs as a function of task demands, stimulation intensity, electrode placement, and number of sessions. With regard to tDCS intensity, non-linear effects of tDCS intensity have been demonstrated, such that increased tDCS intensity does not necessarily equate to increased effectiveness^40,41^. Additionally, the success of varying tDCS intensities is influenced by the population targeted. For example, researchers studying healthy younger adults have reported that lower tDCS intensity (e.g. 1 mA) elicits greater WM gains than higher intensity^31^ whereas other researchers have found that higher intensity (e.g. 2 mA) is necessary to elicit WM gains in clinical populations^42^. In our previous work, we demonstrated that older adults are more similar to clinical populations in their requirement of higher tDCS intensity (e.g. 2 mA) for significant cognitive improvement^12^, but we would not necessarily expect linear improvements with increasingly higher intensities, as the effect of tDCS intensity on WM gains is likely influenced by other factors, especially in aging. At the cellular and network levels, WM training necessarily involves frontoparietal networks^43,44^ interacting with striatal regions^45–48^ and relying on dopamine (DA) signalling^49^. This establishes the rationale for extending work showing that tDCS modulates DA signalling in prefrontal regions via the D1 receptor^50^ and in the striatum, primarily via the D2 receptor^51^. Furthermore, tDCS modulates secretion of brain derived neurotropic factor (BDNF)^52^, a protein which supports memory and learning^53,54^ and perhaps WM^53,55^. Identifying how tDCS-linked WM improvement is influenced by genetic variability within genes that are known to have functional effects on cognitive tasks will enhance our understanding of individual differences following tDCS interventions *and* reveal aspects of the underlying neural mechanism.

Two common single nucleotide polymorphisms significantly modulate DA signalling in WM relevant networks with previous work describing interactions between genotype and tDCS on non-WM tasks (reviewed in^56–59^). The first and most studied polymorphism with regard to WM codes for catechol-o-methyl transferase (COMT), the enzyme primarily responsible for DA metabolism in prefrontal cortex (reviewed in:^60–62^). A common single point mutation (val^158^met) modulates the rate of DA metabolism at the synapse: val/val is rapid, whereas each additional met allele slows enzymatic activity^60,63,64^. This mutation predicts performance on WM and other executive function tasks^65–70^ in a task-dependent manner: val/val participants show greater cognitive flexibility benefiting task switching, whereas met/met homozygotes show enhanced cognitive stability, benefiting maintenance^71^. Age enhances these COMT effects^72–75^, such that val + older adults have worse spatial updating^76^, fluid intelligence, processing speed, and episodic memory compared to met + adults^75^. Furthermore, val + participants have lower baseline WM performance across various tasks but improve more than met/met individuals^77^. Possessing a met allele has been associated with a higher stress response, which in turn leads to reduced WM performance under acute stress^78^. However, one recent study found no WM impairment in met/met carriers, nor did early life stressful and traumatic events correlate with WM performance^79^. Several studies applying 1 mA tDCS to the left dorsolateral prefrontal cortex (PFC) and examining COMT genotype show that met/met carriers exhibit poorer cognitive flexibility following a single session of anodal tDCS^80^, whereas val/val carriers exhibited poorer response inhibition following cathodal tDCS^81^. These findings provide some support for the perspective that genotype does interact with the cognitive effects of tDCS.

A second common polymorphism relevant to WM and WM performance in older adulthood is the number of tandem repeats in a portion of the dopamine transporter (DAT) gene^82^. DAT facilitates dopamine (DA) clearing from the synapse for repackaging into new vesicles and is present in all DA networks throughout the brain^83^. More repeats are associated with higher DAT concentrations, less extracellular DA, and reduced striatal signalling^84^. Variations in DAT regulate pre- and postsynaptic DA concentration^85^, particularly in the striatum^86–89^, and frontostriatal interactions predict WM training gains^48,90–95^.

A third polymorphism determining tDCS response is brain derived neurotropic factor (BDNF val^66^met). Here, there is less accord regarding the effects on WM; BDNF is closely associated with episodic memory^96^, and a lack of BDNF is associated with age-related cognitive decline^97^. Older val/val adults are more susceptible to distractors^98^, and while met + participants performed better at WM tasks^99^, they were worse on tasks of processing speed, delayed recall, and general intelligence^100^. The most relevant recent finding is that anodal tDCS appears to increase BDNF in older adults (reviewed in:^52^) and may differentially facilitate benefits across genotypes.

Collectively, emerging findings suggest that common polymorphisms may influence tDCS effects. Understanding these contributions may elucidate an underlying mechanism of tDCS-linked benefits and may facilitate *a priori* identification of participants who are likely to benefit from tDCS. By combining data from two published WM training + tDCS studies we gained greater statistical power to evaluate whether these three polymorphisms predicted WM training gains^9,12^. Furthermore, these studies parametrically varied tDCS intensity (sham, 1.0 mA, 1.5 mA, or 2.0 mA), enabling us to investigate the dose-dependency of effects and test whether more is better in these WM tasks.

## Results

For all results, Mixed-Method ANOVAs were completed comparing within (WM tasks) and between (tDCS intensity, genotypes) subject factors. All post-hoc pairwise analyses were conducted using Bonferroni corrections for multiple comparisons.

### Day 1 Performance

To determine if genotype (COMT val^158^met, DAT, or BDNF val^66^met) predicted initial tDCS effects on the WM training tasks, Day 1 performance values were subjected to separate Mixed-Method ANOVAs: 2 (Task Type: Visual WM, Spatial WM) × 4 (tDCS intensity: Sham, Active1, Active1.5, Active2) x genotype. In these separate analyses, genotype was either 2 levels (DAT: 9 + DAT, 10 + DAT) or 3 levels (BDNF: val/val, val/met, met/met; COMT: val/val, val/met, met/met). Because neither the BDNF nor the DAT had significant main effects (both F values < 1, *p* values > 0.05) nor any significant interactions at baseline or after training (all p values > 0.17), these analyses are not further discussed. For the breakdown of tDCS Group by COMT genotype, see Table 1.

**Table 1:** TDCS x COMT Groups.

	Val/Val	Val/Met	Met/Met	Total
Sham	8	21	11	40
Active1^	4	15	9	28
Active1.5^+^	4	24	13	41
Active2^	9	14	5	28
Total	25	74	38	

^Indicates 5-day tDCS Protocol; ^+^Indicates 10-day tDCS Protocol.

**Figure 1:**
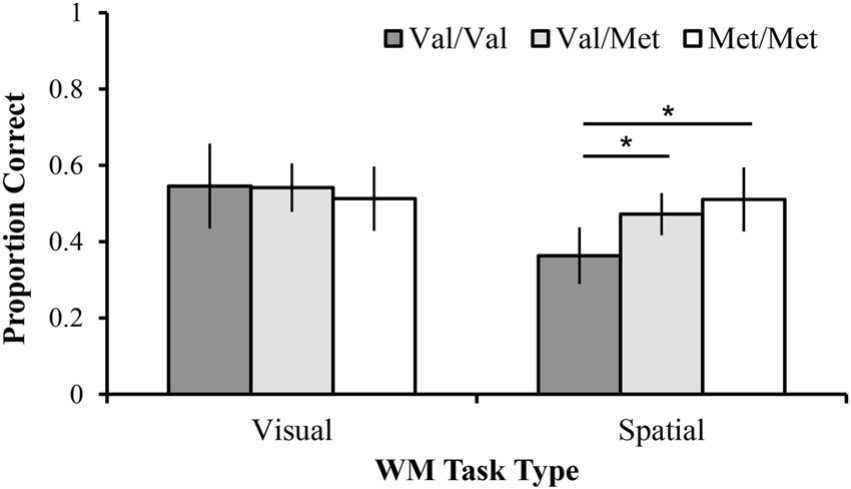
Interaction of COMT and Task Type after initial tDCS session. These data reflect initial responses to a single session of anodal tDCS to frontoparietal sites. The val/val group had significantly poorer initial performance on the Spatial WM task compared to the val/met and met/met groups. *Indicates p < 0.05; Error bars represent standard error of the mean.

Results from a Mixed Method ANOVA (within-subject factor: WM task; between-subject factors: tDCS intensity and COMT genotype) revealed a significant main effect of WM task, such that for all participants, accuracy was higher on the Visual WM task (Mean (M) = 0.53, (Standard Error of the Mean (SE) (0.01)) compared to the Spatial WM task (M = 0.46 (0.01)), F_1,125_ = 13.73, *p* < 0.001, ${\eta }_{p}^{2}=0.10$). The main effect of tDCS intensity approached significance (F_3,125_ = 2.55, *p* = 0.059, ${\eta }_{p}^{2}=0.058$). There was no significant main effect of COMT genotype (F_2,125_ = 2.18, *p* = 0.12). There was, however, a significant COMT genotype x WM Task interaction (F_(2,125)_ = 6.62, *p* = 0.002, ${\eta }_{p}^{2}=0.10$). Bonferroni-corrected pairwise analyses revealed that on the Visual WM task, the COMT val/val (M = 0.55 (0.04)) and val/met (M = 0.54 (0.02)) groups had slightly better accuracy than the met/met group (M = 0.51 (0.03)), although these performance differences were not significant, *p values* > *0.41*. Significant differences were observed on the Spatial WM task. The val/val group (M = 0.36 (0.03)) had significantly poorer accuracy than the val/met group (M = 0.47 (0.02)), *p* = 0.001 and the met/met group (M = 0.51 (0.03)), *p* < 0.001; see Fig. 1. No significant differences existed between the val/met group and the met/met group, *p* = 0.18. No other interactions approached significance (all p’s > 0.18).

### Follow-up Accuracy

To identify interactions between longitudinal tDCS and genotype, a second set of analyses examined the change in performance from Day 1 to follow-up one month after the end of training and tDCS. Again, because neither BDNF nor DAT showed significant main effects or interactions, these analyses are not discussed.

**Figure 2:**
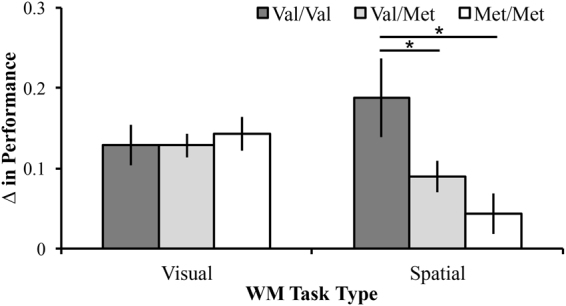
Interaction of COMT and Task Type on Change in Performance. These data reflect the the normalized difference score in performance from baseline after completion of tDCS and WM training after 1 month of no contact. The val/val group had a significantly greater change in performance than the val/met and met/met groups. *Indicates p < 0.05; Error bars represent standard error of the mean.

Results from a Mixed Method ANOVA (within-subject factor: WM task; between-subject factors: tDCS intensity and COMT genotype) found no significant main effects of tDCS intensity (F_3,125_ = 1.75, *p* = 0.16), or WM Task (F_1,125_ = 2.22, *p* = 0.14). There was a significant main effect of COMT genotype (F_2,125_ = 4.44, *p* = 0.01, ${\eta }_{p}^{2}=0.07$). Across tasks, the COMT val/val group (M = 0.18 (0.02)) showed significantly greater improvement than the val/met group (M = 0.11, (0.01)), *p* = 0.004 and the met/met group (M = 0.09 (0.02)), *p* = 0.01). No differences existed between the val/met group and the met/met group, *p* = 0.38. There was also a significant COMT genotype x WM task interaction (F_2,125_ = 5.14, *p* = 0.007, ${\eta }_{p}^{2}=0.08$). Pairwise comparisons revealed that, on the Visual WM task, the val/val group (M = 0.13 (0.03)) and val/met group (M = 0.13, (0.02)) had less improvement than the met/met group (M = 0.14, (0.02)), but these differences did not reach statistical significance, *p* values > 0.68. However, there were significant between group differences on the Spatial WM task. The val/val group (M = 0.21, (0.04)) showed significantly greater improvement than the val/met group (M = 0.09, (0.02)), *p* = 0.006 and the met/met group (M = 0.02 (0.03)), *p* < 0.001. No significant differences existed between the val/met group and met/met group, *p* = 0.11; see Fig. 2. Although group differences were not significant on the Visual WM task, participants tended to show greater improvement in areas where they had been weakest. Importantly, because the WM tasks were difficult, performance was not affected by ceiling effects (overall accuracy across participants on the Visual WM task was 68.8% (SD: 0.17) and 55.6% (SD: 0.17) on the Spatial WM task).

Of greatest relevance, there was a significant 3-way interaction of tDCS intensity x COMT genotype x WM Task (F_6,125_ = 2.39, *p* = 0.03, ${\eta }_{p}^{2}=0.10$) highlighting dose-dependent tDCS effects. To understand this complicated interaction, we examined change in performance as a function of tDCS intensity for each COMT genotype.

**Figure 3:**
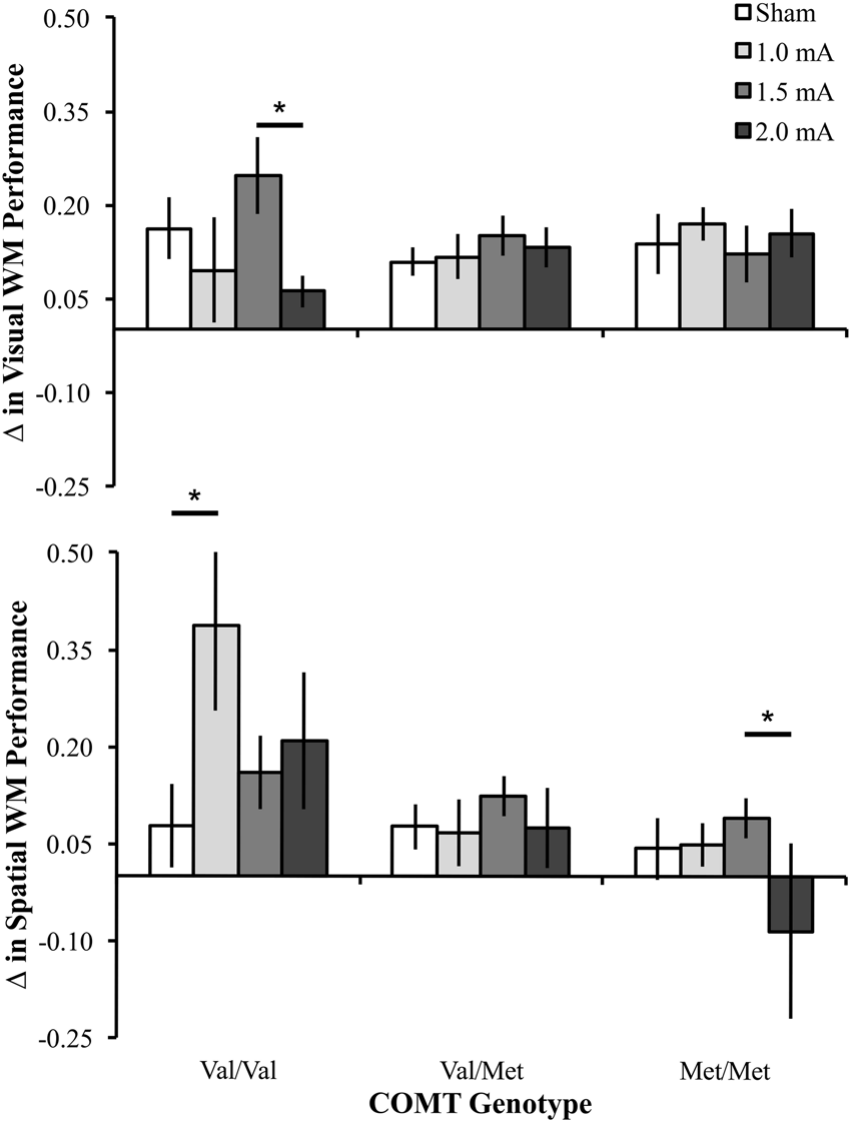
Interaction of COMT on Change in Performance with tDCS Subgroups. On the Visual WM task, Val/val participants in the Active 1.5 tDCS group had a significantly greater change in performance than the Active 2 group. On the Spatial WM task, val/val participants in the Active 1 tDCS group had a significantly greater change in performance than the Sham group. On the Spatial WM task, met/met participants in the Active 1.5 tDCS group had a significantly greater change in performance than the Active 2 tDCS group, whose performance was poorer at follow-up than baseline. *Indicates p < 0.05; Error bars represent standard error of the mean.

On the Visual WM task, in the val/val group, the Active 1.5 tDCS group (M = 0.25 (0.02)) had significantly greater gains than the Active 2 tDCS group (M = 0.06 (0.04)), *p* = 0.01. No other significant differences existed within the homozygous val/val group between tDCS groups, *p* values > 0.07. Additionally, no significant differences existed within the val/met group between tDCS groups, *p* values > 0.27 nor within the met/met group, *p* values > 0.42. On the Spatial WM task, within the val/val group, the Active 1 tDCS group (M = 0.39 (0.12)) group had significantly greater gains than the Sham tDCS group (M = 0.08 (0.06), *p* = 0.04). No other significant differences existed within the val/val group between tDCS groups, *p* values > 0.18. No significant differences existed within the val/met group between tDCS groups, *p* values > 0.33. Within the met/met group, the Active 1.5 tDCS group (M = 0.09 (0.04)) had significantly greater improvement than the Active 2 tDCS group (M = −0.08 (0.07)), *p* = 0.03. No other significant differences existed within the met/met group between tDCS groups, *p* values > 0.12; see Fig. 3.

We also examined change in performance as a function of COMT genotype in each tDCS group. On the Visual WM task, no significant differences existed between COMT groups within the Sham tDCS group, *p* values > 0.30, within the Active 1 tDCS group, *p* values > 0.32, within the Active 1.5 tDCS group, *p* values > 0.16, or within the Active 2 tDCS group, *p* values > 0.10. On the Spatial WM task, no significant differences existed between the COMT groups within the Sham tDCS group, *p* values > 0.58 or within the Active 1.5 tDCS group, *p* values > 0.36. Significant differences did exist within the Active 1 tDCS group. The val/val group (M = 0.39, (0.09)) had significantly greater improvement than the val/met group (M = 0.07, (0.05)), *p* = 0.003 and met/met group (M = 0.05 (0.06)), *p* = 0.003; see Fig. 3B. No differences existed within the Active 1 group between the val/met and met/met group, *p* = 0.79. There were also group differences within the Active 2 tDCS group. The val/val group (M = 0.21 (0.09)) had significantly greater gains than the met/met group (M = −0.08, (0.12)), *p* = 0.05. No other significant differences existed within the Active 2 group between the COMT groups within the Active 2 tDCS group, *p* values > 0.23.

## Discussion

Older adults are interested in stabilizing or improving their cognitive ability. Non-invasive neurostimulation, including tDCS, is a welcome contender to achieve this goal. However, there is an incomplete understanding of the mechanisms underlying tDCS effects and unknown factors that may predict immediate and lasting tDCS effects. Here, we reported new analyses using data from two longitudinal studies that paired WM training with anodal frontoparietal tDCS in healthy older adults. We hypothesized that genetic polymorphisms - COMT val^66^met, DAT, and BDNF val^66^met – might predict the initial or the long-term effects of tDCS on visual and spatial WM. The results demonstrated no discernible effect of DAT or BDNF val^66^met but indicated that COMT val^66^met may influence the effects of WM training and tDCS. Interestingly, COMT did not have a simple influence, where one variation was optimal. Instead, COMT predicted initial WM performance after a single session of anodal frontoparietal tDCS and appeared to predict the nature of training related gains. Generally, each COMT group improved on a WM task where they needed improvement, although gains were higher on the Spatial WM task – a more challenging task for these participants. Perhaps more surprisingly, these COMT effects appeared to depend on tDCS intensity. At follow-up, the val/val group benefited most from intermediate intensity (1.5 mA) tDCS on Visual WM tasks but benefited most from lower intensity tDCS (1 mA) on Spatial WM tasks. The met/met group responded differently; high intensity tDCS (2 mA) elicited significantly poorer performance on the Spatial WM task compared to intermediate intensity (1.5 mA). Because there is a relationship between the COMT status and the effects of longitudinal tDCS paired with WM training, we interpret the findings as supporting the involvement of prefrontal DA signalling in the underlying tDCS-linked neuroplastic mechanism. They also indicate that more distal effects on DA in striatal regions via DAT, or effects on BDNF are less likely to account for tDCS-linked WM benefits.

Previous findings propose that ideal prefrontal DA levels are intermediate and follow an inverted U-shaped function^49^. Some of these data follow that prediction. On the Visual WM task the COMT val/val participants showed their greatest gains after intermediate intensity tDCS; see Fig. 3. However, this did not occur in val/val group where low tDCS intensity (1 mA) prompted the greatest gains on the Spatial WM task. Thus, the U-shaped function is difficult to fit to each genotype to select the ideal tDCS intensity across WM tasks. Presumably other factors, genetic or paradigmatic, contribute to tDCS effect on complex cognitive tasks like WM. These factors increase dopaminergic levels in the PFC, which in turn likely interact to a greater extent with the effects of tDCS to this region. The current findings support further investigation to eventually develop a clear relationship between tDCS intensity and task relevant genotypes. Achieving this ambitious goal requires more scientific inquiry, including how the training task and cognitive domain influence results. Furthermore, these data highlight the need for greater demographic collection in tDCS studies, as not all participants respond similarly to the same protocol^32,34,101^.

*Why* and *how* tDCS impacts WM performance matters. Although the effect of tDCS is mostly predictable in motor behaviour paradigms (reviewed in:^102^), the effect on higher order cognitive processes is more convoluted. Executive function tasks, like WM, require many regions working together (e.g., frontoparietal network for WM). More information is essential for determining how to optimize tDCS-based interventions for individuals. There is likely a role of COMT genotype in predicting tDCS-linked WM gains, yet this role is difficult to describe simply. A conservative approach may be to apply an intermediate dose of tDCS (1.5 mA), but it remains possible that gains in one domain (e.g. Visual WM) come with losses in another (e.g. Spatial WM). These data suggest that uniform tDCS parameters, such as current intensity, are not appropriate. The existence of a do-it-yourself tDCS community, individuals who self-administer commercial products or homemade tDCS devices to benefit from these nootropic therapies may be disappointed, but a Supercharged Brain is unlikely. Only with more research can we elucidate the full range of gains and losses across individuals and accurately recommend a tailor-made tDCS intervention.

One challenge in exploring genetic contributions to cognition is the need for a larger number of participants to achieve adequate power. Despite combining data from two studies with large sample sizes, several cells had very low N. Although this limitation reduced the generalizability of the present findings, it nevertheless provided provocative support for future investigation. We acknowledge that with additional power, a contribution of DAT and BDNF might emerge. To address this, future studies should consider replicating these methods with larger N or conducting genotyping across multiple research groups of tDCS researchers to accumulate sufficient data while being cost-effective. A related limitation is associated with statistical analysis of uneven and small tDCS x COMT groups. Although the use of parametric statistics does not necessarily violate statistical assumptions, it is a conservative approach that makes detection of significant differences between small groups challenging, and there is not a universally agreed-upon non-parametric analogue. Given low power, when we corrected for multiple comparisons, we saw limited differences between the COMT polymorphisms as a function of tDCS intensity. A substantially increased sample size may reveal additional tDCS intensity and COMT genotype interactions that we had insufficient power to detect. As an alternative statistical approach, we examined our data by subjecting the follow-up − baseline normalized difference scores ((follow-up − baseline)/(follow-up + baseline)) to a univariate Analysis of Co-variance (ANCOVA) with baseline performance as the covariate. This alternative approach was generally consistent with the results we described – which improved our confidence in the findings - but it is not consistent with how we previously analysed. Overall, we acknowledge that the small tDCS x COMT group sizes within our study render our findings as an early step in understanding the mechanism behind tDCS-linked WM training benefits. Subsequently, we remain tentative about our conclusions. Our hypotheses were exploratory and challenging to test, especially given the substantial cost and time necessary for adequate statistical power. Nevertheless, these results provide important insight into one of factors that should be included in future study designs to either mitigate or explain inter-subject variability in tDCS-linked responsiveness.

A third limitation of this work is that we tested one population: healthy older adults. Because aging induces grey matter loss, degrades white matter integrity^103–105^, and influences the gene expression^73,75,76,82,97,100^, may mean the aging population is not representative. Again, replication in a more diverse age range would better clarify how age predicts outcomes. Finally, the two studies we combined included slightly varied tDCS protocols. The participants completed different durations of tDCS + WM training following slightly different tDCS protocols. Finally, one subgroup received tDCS to PPC only without frontal lobe stimulation. Although tDCS affects large cortical regions, it is a reasonable criticism that the mechanism we propose highlights prefrontal activity, when in this subgroup we did not target PFC. However, based on the results of that study^9^, we propose that stimulation of any node of the frontoparietal network, likely influences the entire network. Nevertheless, by pairing slightly different studies, we improved our statistical power to explore our hypotheses. We acknowledge that better consistency with the protocol duration would strengthen our methodology and interpretation of findings. Importantly, however, future work can build upon these preliminary findings to confirm and clarify the role of frontal and parietal regions in tDCS-linked WM improvement and continue to optimize tDCS protocols. We note that tDCS may enhance empirically designed brain training^106^, and exercise^107^ as a beneficial approach for countering other age-related concerns such as cortical disconnection^108^, or reduced connectivity^109^. TDCS may thus serve to facilitate neuroplastic changes in the brain. However, more work is needed to clarify how to maximize cognitive benefits in each participant.

A conservative message for those interested in tDCS is: *tDCS is complicated*. Merely placing electrodes on the head and supplying current may not elicit positive results. There are influences of task demands, individual differences (education, age, anatomy, genotype, level of fatigue, diet, others yet to be considered), and stimulation parameters (duration, intensity, location) that must be better researched and understood before tDCS effects can be reliably predicted ahead of use. The weight that each of these influences affects the tDCS-linked cognitive benefits is also still unknown; however, the present results shed light on one likely mechanism, participant genotype, which may be responsible for some of the previously reported inconsistent or null tDCS findings.

## Conclusion

Here, we demonstrated the influences of tDCS intensity, COMT genotype, and task demands on tDCS-linked WM training gains. Specifically, our results suggest PFC tDCS amplitude disproportionally affects those with different COMT genotypes based on task demands. This trend may be one of many which influence the variable effects reported in the neurostimulation literature specific to cognitive tasks. Despite the small sample size for some of the genotype and amplitude cells, we observed the largest effect in those with the val/val COMT polymorphism, where 1.5 mA of tDCS improved performance on Visual WM tasks, whereas 1 mA had the greatest improvement on Spatial WM tasks. These U-shaped dose-response findings were most apparent in the COMT val/val genotype; however, the same pattern was observed in the opposite direction for the COMT met/met genotype. These findings point toward a future where an individual’s genotype may play a role in specifying an appropriate tDCS-linked cognitive intervention. For WM, these data demonstrate that tDCS to frontoparietal networks likely relies on titrating DA. More studies are needed to identify who is likely to benefit from any tDCS protocol on a per task domain basis. Continued research exploring these influences findings may allow researchers and clinicians to capitalize on translational use of tDCS in cognitive maintenance or enhancement via interventions tailored for individual needs and characteristics.

## Methods

### Participants

Data were collected from 146 participants from a 10-day^9^ and a 5-day^12^ tDCS + WM training study. The 10-day study included 72 neurotypical right-handed older adult participants (mean age: 64.27; SD: 5.15; 38 female); 57 of whom provided viable DNA samples^9^. The 5-day study included 90 neurotypical right-handed older adults (mean age: 69.03; SD: 8.63; 48 female), and 89 participants provided usable DNA samples^12^. To ensure that assumptions were met for our statistical analyses, nine outliers were removed based on floor (<10%: N = 7) or ceiling (>90%: N = 2) baseline performance. 137 participants are included in analyses. No significant differences were observed between tDCS, DAT, COMT or BDNF groups in age or years of education (*p* values > 0.05).

### 10-Day and 5-Day Study Differences

**Figure 4:**
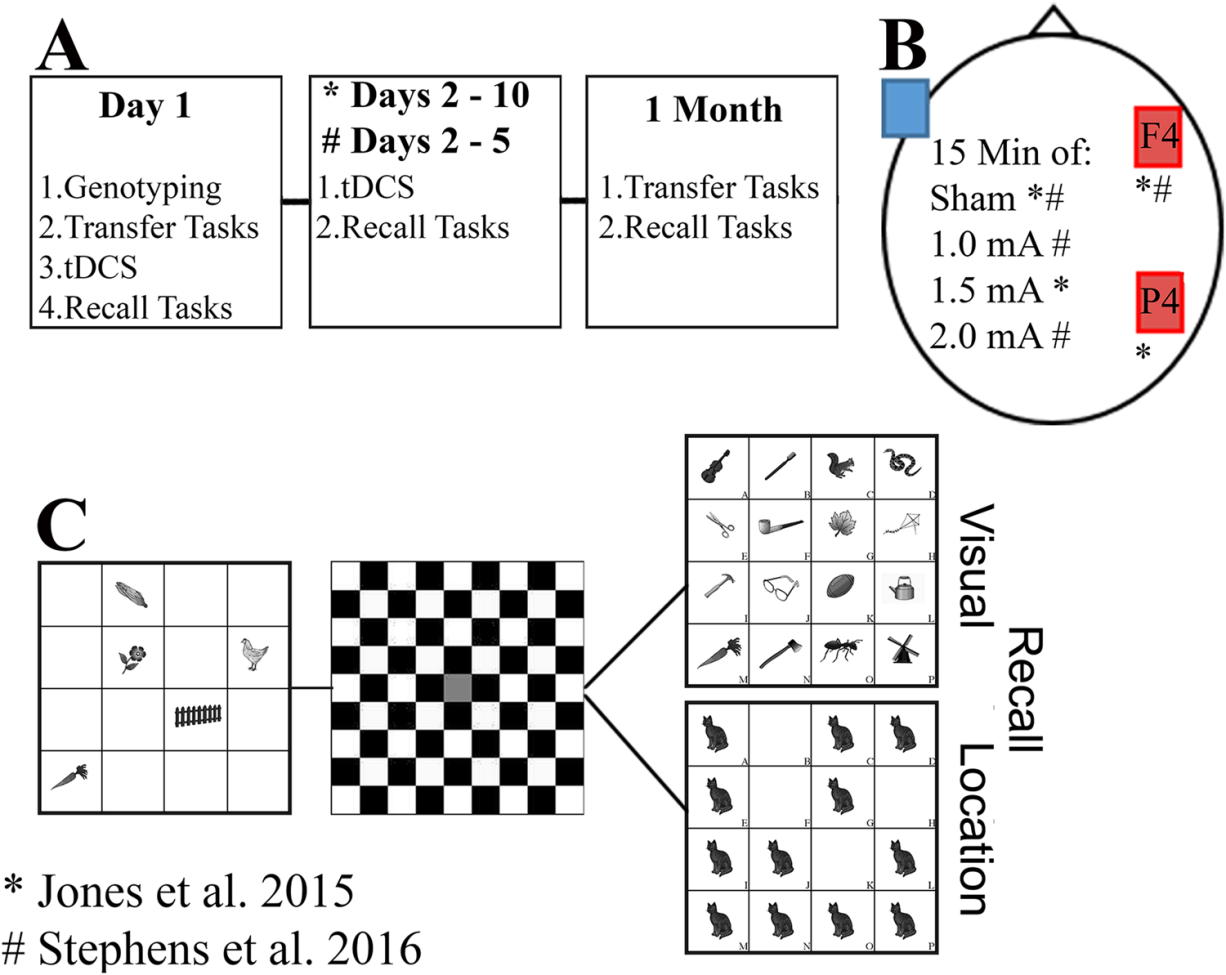
WM Training Paradigms and tDCS Parameters. The WM training and tDCS parameters for each of the two studies (for more see the methods section of each study). The ^*^symbol represents aspects of the paradigm for the Jones *et al*. study, and ^#^represents aspects of the paradigm for the Stephens *et al*. study. (**A**) The order of WM training for the two studies. (**B**) The tDCS montage used for the two studies, the anode was located over F4 or P4 with the cathode reference electrode placed over the left cheek. (**C**) The WM training paradigm for the tasks that overlapped between the studies. The visuospatial recall task, five items appeared (3° visual angle, 200 ms) followed by a delay period filled with a checkerboard (4000 ms), after which 12 images appeared. Participants selected which locations were filled during the encoding period. In the verbal recall task, five items appeared (3° visual angle, 2000 ms) followed by a delay period filled with a checkerboard (500 ms), after which participants selected the old item from among 16 choices.

There were some differences between the two studies (for full details, see:^9,12^). The 10-day study involved training over 10 consecutive weekdays, whereas the 5-day study was completed over 5 consecutive weekdays (see Fig. 4). Participants completed the same WM training tasks and genotyping procedure, but tDCS protocols differed (see tDCS Administration). All experimental protocols were carried out in accordance with the Declaration of Helsinki and with the recommendations of the University of Nevada’s Institutional Review Board. The University of Nevada’s Institutional Review Board approved all study procedures. All subjects provided informed written consent and received $15/hour.

### WM Training Tasks

Each 45-minute training session immediately followed tDCS. Participants performed a Spatial and Visual recall WM task (see:^9,12^ for further paradigm details; Fig. 4). In the Spatial WM task, 5 images (e.g. corn, flower, fence, carrot, chicken; 3° visual angle) were presented (200 ms) in 5 of 16 possible locations. After a delay (4000 ms), 12 images appeared. Participants selected the 1 location that was occupied during the initial presentation. This task is more accurately a ‘visuospatial’ task, which requires participants to track spatial locations with visual input. To differentiate it from the Visual WM task, we refer it to it as the Spatial WM task. In the Visual WM task, 5 items were presented (2000 ms) and after a delay (500 ms), 16 items appeared. Participants selected the 1 item that was repeated from the initial stimulus array. Both tasks were un-speeded. Participants completed 2 blocks of 25 trials per task; tasks were presented in a counterbalanced order. The primary outcome measures were accuracy on Day 1 (i.e. baseline) and a calculated change in performance through normalized difference scores ((Follow-up accuracy − Day 1 accuracy)/(Follow-up accuracy + Day 1 accuracy)). This calculation has precedence in other WM training studies^9,110–112^. Note that these are WM tasks probed by recall, making chance performance less than 50%.

### Genotyping Procedure

DNA samples were genotyped in an off-site commercial laboratory (GenoTek Labs, United States) using standard procedures (http://www.dnagenotek.com/US/genomicservices/genofind.html ^)^. Two single nucleotide polymorphisms, COMT val^158^met (rs4680: val/val, val/met, met/met) and BDNF val^66^met (rs6265: val/val, val/met, met/met), and DAT variable number tandem repeat (VNTR), DAT1 (SLC6A: 9/9 or 9/10 Repeats (9 + DAT), 10/10 or 10/11 Repeats (10 + DAT)) were analysed. The DAT groupings were completed by grouping 9/9 and 9/10 together and 10/10 and 10/11 to form a 9 + DAT and 10 + DAT groups, as few participants were 9 allele homozygous (4 participants) and only 1 participant was 10/11. This is consistent with other analyses in the DAT literature^84^.The observed genotype frequencies were found to be consistent with the Hardy-Weinberg equilibrium (COMT: χ^2^ = 3.34; *p* > .05; val/val = 25, val/met = 74, met/met = 38; BDNF: χ^2^ = 1.79; *p* > .05; val/val = 84, val/met = 46, met/met = 7; DAT: χ^2^ = 2.65; *p* > .05; 9DAT = 67, 10DAT = 70). Participants were arrayed among the tDCS groups as follows: Sham (N = 40), Active1 (N = 28), Active1.5 (N = 41), Active2 (N = 28).

### TDCS Administration

The neuroConn tDCS device provided stimulation (Eldith MagSteim, GmbH, Ilmenau, Germany). Current was delivered through two 5 × 7 cm electrodes encased in saline-soaked sponges; the reference electrode was placed on the contralateral cheek^33,113–118^. Participants were blind to their tDCS condition. In the 10-day study, participants received 10 minutes of 1.5 mA (Active 1.5) anodal tDCS to a) the right PFC (F4), b) right posterior parietal cortex (PPC: P4), or c) stimulation altered between PFC and PPC sites^119^ while completing practice trials of the WM training tasks. This created four groups of 18 participants in each of the four conditions. As there were no between group behavioural differences (*p* = 0.74), data were collapsed across stimulation site to form the ‘Active 1.5’ group. In the 5-day study, participants received sham or 15 minutes of anodal tDCS to the right PFC (F4) while completing practice trials of the WM training tasks. Thirty participants received 1 mA (Active 1), thirty participants received 2 mA (Active 2), and thirty participants received only sham tDCS. For both studies, the tDCS group sizes represent all included participants and exclude the participants who did not provide DNA samples or those who were excluded for floor or ceiling effects.

### Data Availability

The data reported in this study are included in the Supplementary Information files.

## Electronic supplementary material

Supplementary Figures
