# Supplementary material for: Task demands, tDCS intensity, and the COMT val158met polymorphism impact tDCS-linked working memory training gains
Source: Sci Rep. 2017 Oct 18;7:13463. doi: 10.1038/s41598-017-14030-7 (PMC5647397; doi:10.1038/s41598-017-14030-7)
Supplement: Supplementary file 1 — Supplementary Figures [file 41598_2017_14030_MOESM1_ESM.doc]

**Task demands, tDCS intensity, and the COMT val^158^met polymorphism impact tDCS-linked working memory training gains**

Jaclyn A. Stephens, Kevin T. Jones, Marian E. Berryhill

**
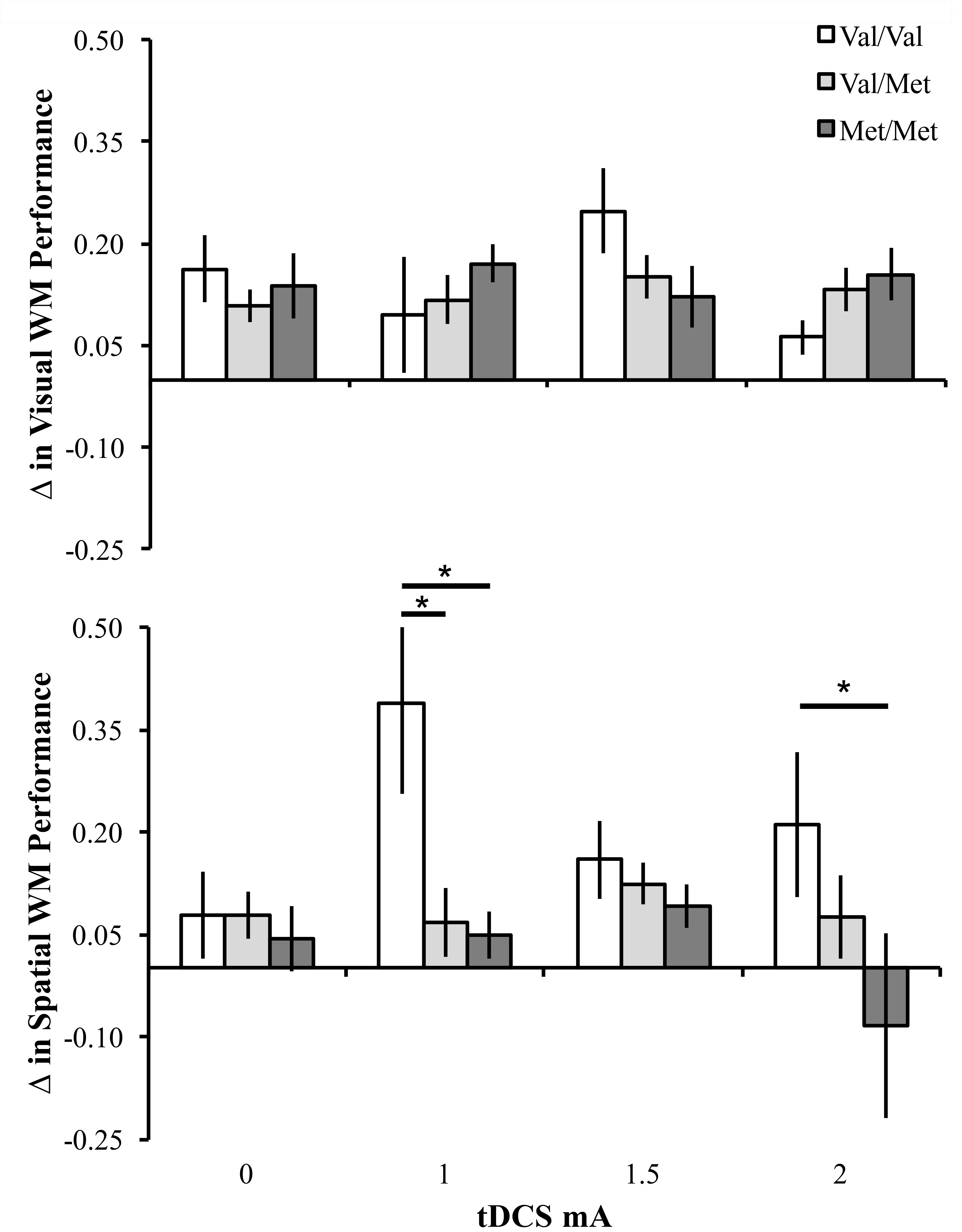
**

**Supplementary Figure 1**

Interaction of tDCS on Change in Performance with COMT Subgroups

On the Visual WM task, there were no differences within any tDCS groups between varying COMT genotypes. On the Spatial WM task, Active 1 participants who were val/val homozygotes had a significantly greater change in performance than met/met homozygotes. On the Spatial WM task, Active 2 participants who were val/val homozygotes had a significantly greater change in performance than met/met homozygotes, whose performance was poorer at follow-up than baseline. This is the same data plotted in Figure 3, however with tDCS on the x-axis as opposed to COMT status.

* indicates p <. 05; Error bars represent standard error of the mean.


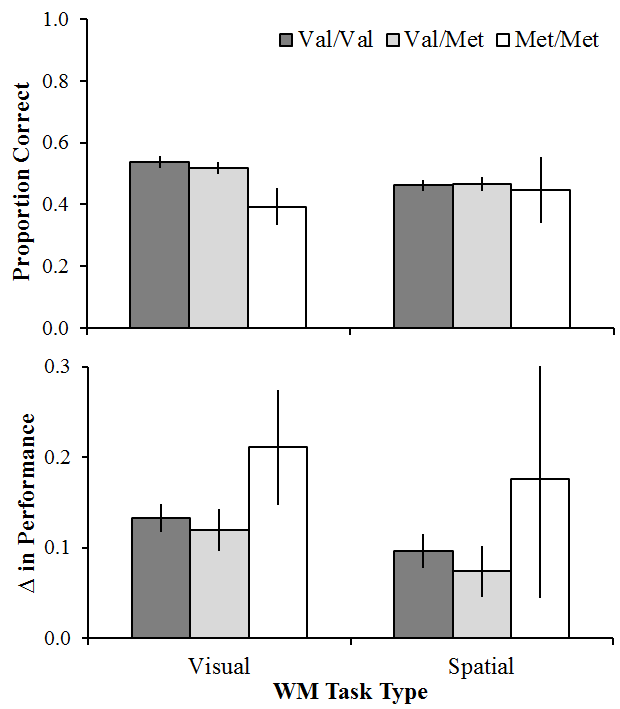


**Supplementary Figure 2**

**BDNF Genotype Accuracy at Baseline and Change in Performance After WM Training.**

**Top**) Accuracy at baseline based on BDNF status. **Bottom**) Change in accuracy as measured by normalized difference scores from baseline based on BDNF status. The met/met group contains only four participants.


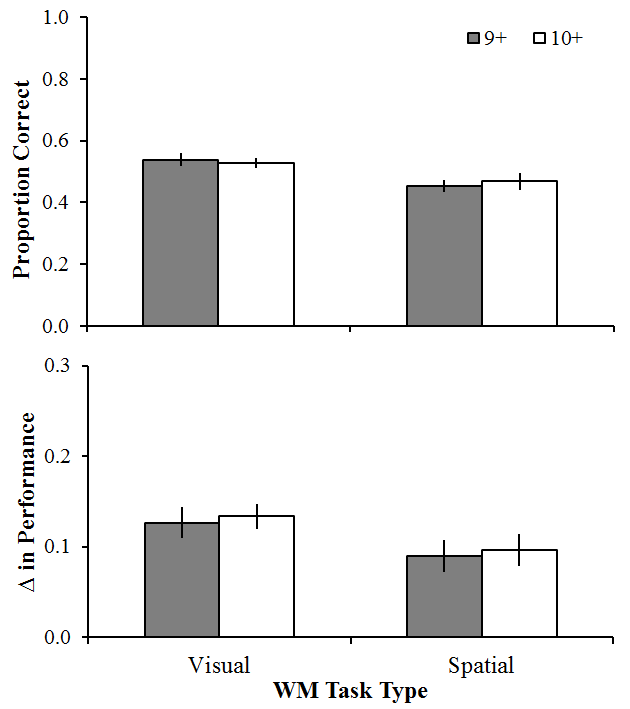


**Supplementary Figure 3**

**DAT Genotype Accuracy at Baseline and Change in Performance After WM Training.**

**Top**) Accuracy at baseline based on DAT status. **Bottom**) Change in accuracy as measured by normalized difference scores from baseline based on DAT status. Two DAT groups were combined as they both contain a 9 repetition. Furthermore, the sample of DAT1 was only four participants split across different tDCS conditions. The DAT4 group was combined with the DAT3 group due to the 10 repetition, and a simple size of only one participant in the DAT4 group.

**
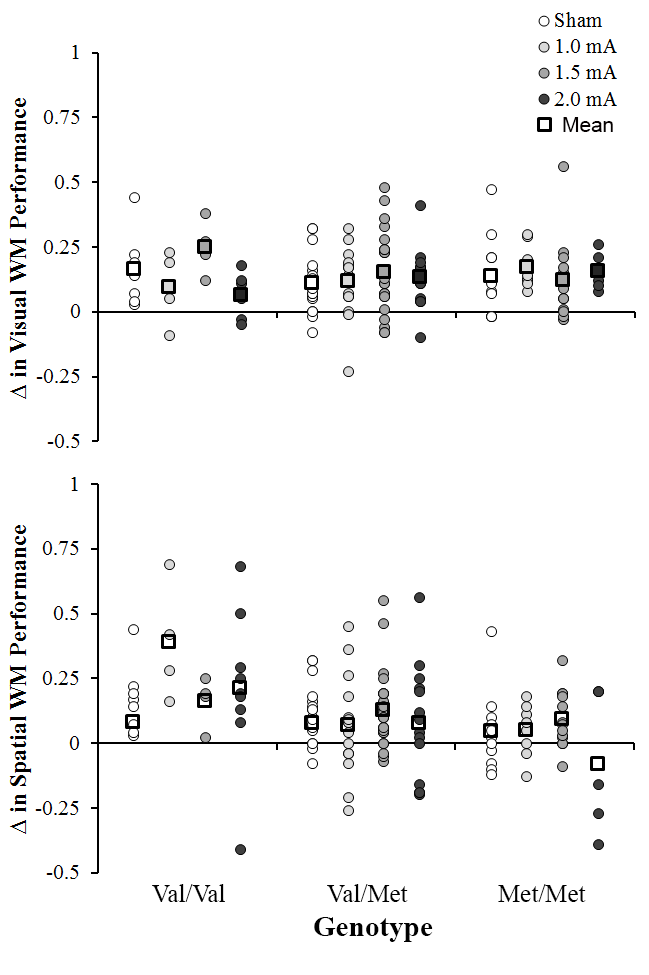
**

**Supplementary Figure 4**

**Individual Data Normalized Difference Scores Following Training Based on COMT Allele**

**Top**) Difference in accuracy as measured by Normalized accuracy difference scores from baseline on the Visual WM task for each of the COMT groups. **Bottom**) Difference in accuracy as measured by Normalized accuracy difference scores from baseline on the Spatial WM task for each of the COMT groups.

**
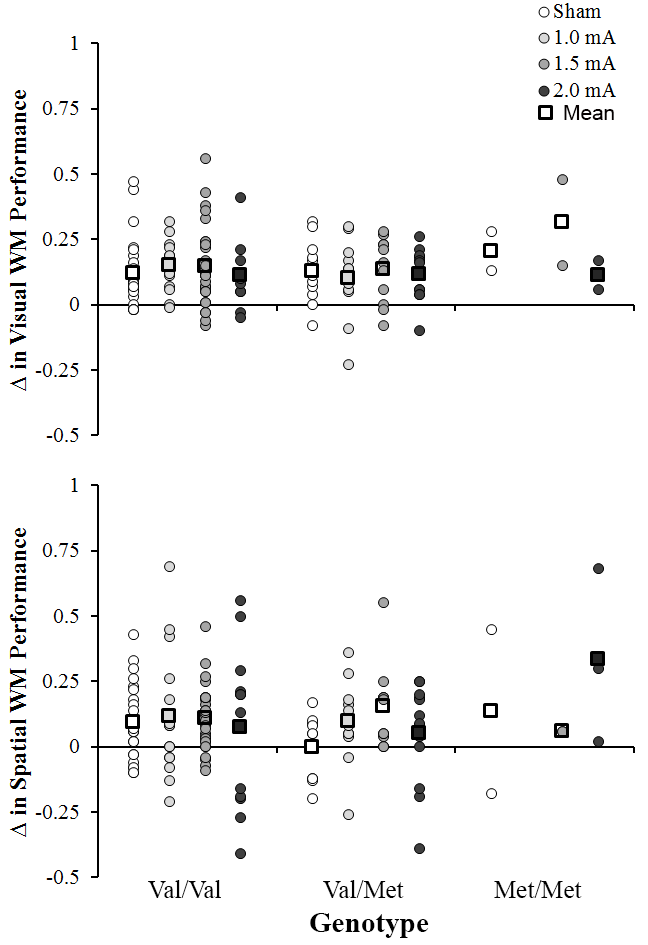
**

**Supplementary Figure 5**

**Individual Data Normalized Difference Scores Following Training Based on BDNF Allele**

**Top**) Difference in accuracy as measured by Normalized accuracy difference scores from baseline on the Visual WM task for each of the BDNF groups. **Bottom**) Difference in accuracy as measured by Normalized accuracy difference scores from baseline on the Spatial WM task for each of the BDNF groups. Note: The met/met allele had zero participants in the 1.0 mA tDCS group.

**
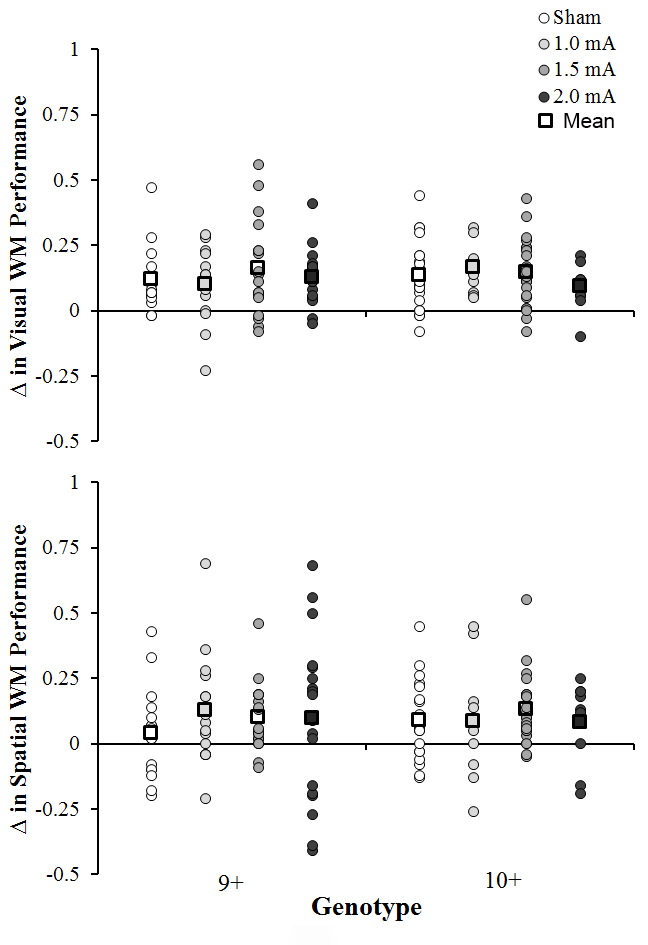
**

**Supplementary Figure 6**

**Individual Data Normalized Difference Scores Following Training Based on DAT Allele**

**Top**) Difference in accuracy as measured by Normalized accuracy difference scores from baseline on the Visual WM task for each of the DAT groups. **Bottom**) Difference in accuracy as measured by Normalized accuracy difference scores from baseline on the Spatial WM task for each of the DAT groups.
